# Urodynamics mixed type urinary incontinence with advanced pelvic organ prolapse, management and outcomes

**DOI:** 10.1038/s41598-020-58594-3

**Published:** 2020-02-06

**Authors:** Tsia-Shu Lo, Ma. Clarissa Uy-Patrimonio, Chuan Chi Kao, Sandy Chua, Ting-Xuan Huang, Ming-Ping Wu

**Affiliations:** 1 Division of Urogynecology, Department of Obstetrics and Gynecology, Chang Gung Memorial Hospital, Linkou Medical Center, Taoyuan, Taiwan Republic of China; 2 Department of Obstetrics and Gynecology, Chang Gung Memorial Hospital, Keelung Medical Center, Keelung, Taiwan Republic of China; 3 Chang Gung University, School of Medicine, Taoyuan, Taiwan Republic of China; 4 Department of Obstetrics and Gynecology, Dr. Pablo O. Torre Memorial Hospital, Bacolod City, Philippines; 5 Department of Obstetrics and Gynecology, Cebu Velez General Hospital, Cebu City, Philippines; 6 Division of Urogynecology and Pelvic Floor Reconstruction, Department of Obstetrics and Gynecology, Chi Mei Foundation Hospital, Tainan, Taiwan; 7 Center of General Education, Chia Nan University of Pharmacy and Science, Tainan, Taiwan

**Keywords:** Urological manifestations, Urinary incontinence

## Abstract

Patients with pelvic organ prolapse (POP) often have accompanying lower urinary tract symptoms. Symptoms such as stress urinary incontinence(SUI-_UD_) and detrusor overactivty(DO) would co-exist in a number of patients. Management entails relieving the obstructive element. To determine the clinical outcome of patients with urodynamics mixed type urinary incontinence(MUI-U) after vaginal pelvic reconstructive surgery(PRS), a retrospective study was conducted. MUI-U was defined as having urodynamic findings of both of DO/DOI (derusor overactivity incontinence) and SUI-_UD_. Main outcome measures: Objective cure- absence of involuntary detrusor contraction on filling cystometry and no demonstrable leakage of urine during increased abdominal pressure; Subjective cure- assessment index score of <1 on UDI-6 question #2 and #3. Of the 82 patients evaluated, 14 underwent vaginal PRS with concomitant mid-urethral sling(MUS) insertion while 68 had vaginal PRS alone. Pre-operatively, 49(60%) patients had stage III and 33(40%) had stage IV prolapse. Post-operatively, 1-year data shows an objective cure of 56% (46/82) and subjective cure of 54% (44/82). MUI-U was significantly improved. Improvement of SUI_UD_ and results of the 1-hour pad test were more pronounced in patients with concomitant MUS insertion. Ergo, vaginal PRS cures symptoms of MUI-U in >50% of patients and concomitant MUS can be offered to SUI predominant MUI.

## Introduction

Mixed urinary incontinence (MUI) alone has been the leading cause of urinary incontinence in women above 65 years old. The ten-year cumulative incidence of urinary incontinence rates MUI as the first reported symptom in 37.2% of elderly patients^1^. The International Urogynecological Association (IUGA) and International Continence Society (ICS) define MUI as the complaint of involuntary leakage of urine associated with exertion, sneezing, or coughing, as well as leakage associated with urgency^2^. The cause of which is due to striated muscle atrophy, estrogen deficiency, abnormalities in histomorphology, and microstructural changes^3^. Diagnosis of MUI through urodynamic studies pose a great challenge, since results fail to correlate with patient’s symptoms, which lead to under diagnosis. Management of these group patients has generally been based on the predominant symptom that the patients report as the most bothersome^4^.

On the other hand, patients with pelvic organ prolapse (POP) often have lower urinary tract symptoms (LUTS). The use of urodynamic study for pre-operative evaluation of patients with POP becomes mandatory per recommendation by International Consultation on Incontinence^5^. Urodynamic studies unmask occult stress urinary incontinence (SUI-_UD_) and identify women with concomitant detrusor overactivity (DO) and overt SUI-_UD_. Women with DO and SUI-_UD_ are considered to have mixed type urinary incontinence (MUI-U). The incidence of MUI in patients with POP is 34.3%^6^. Relieving the obstructive element becomes the main focus of management for these patients since anatomical distortion is the basis for symptomatology. However, SUI and POP outcomes does not just focused on the relief of symptoms but also the patient’s sexual function and quality of life^7,8^

There is a paucity of literature describing the outcome of patients with MUI-U and POP after vaginal pelvic reconstructive surgery (PRS) despite of studies made focusing on the outcome of SUI and POP or DO and POP after vaginal PRS. Hence, the study focuses on the clinical outcome of patients with MUI-U and POP after vaginal PRS. It is hypothesized that vaginal PRS with or without concomitant mid-urethral sling (MUS) insertion has a positive effect on patient symptomatology and cure rates.

## Materials and Methods

A retrospective observational study was conducted in a tertiary referral center from January 2006 to December 2015. Institutional Review Board approval (IRB no. 201800076B0) was obtained from Chang Gung Memorial Hospital Ethics Board Committee. Procedures were done in accordance with the guidelines and regulations of the institution. All patients signed the written informed consent and agreed to the procedures that were performed. The medical records of women who had vaginal PRS for advanced POP (≥Stage 3) with urodynamic diagnosis of mixed type urinary incontinence (MUI-U) - DO and SUI-_UD_ - were gathered. Women with incomplete data, no preoperative urodynamic study, with symptomatic complaints of MUI- OAB and SUI- not reflected on urodynamic study were excluded.

The pre-operative evaluation was comprehensive and followed the institutional protocol. It included medical history, physical exam, pelvic exam, urinalysis, multichannel urodynamic testing, and 3-day voiding diary. Hematuria is infrequently encountered in patients with prolapse as urinalysis was a routine in our practice. If present, we followed a hematuria protocol for disease differentiation. Validated subjective questionnaires such as Urinary Distress Inventory Questionnaire (UDI-6), Incontinence Impact Questionnaire (IIQ-7), Pelvic organ prolapse Distress Inventory (POPDI-6) and Pelvic Organ Prolapse/Urinary incontinence sexual questionnaire (PISQ-12) were answered as well. POP was staged according to the POP-Q system and assessed the patient in semi-lithotomy position. The multichannel urodynamic study was conducted by a trained nurse following the standardized protocol set by the ICS^9^, using the Dantec Menuet System (Dantec Medical A/S, Skovlunde, Denmark) and the Solar Gold system (Medical Measurement Systems, Dover, NH, USA). To diagnose occult SUI during urodynamic study, an appropriately sized pessary was inserted to reduce the prolapse. However, upon measurement of pressure flow, the prolapse was not reduced. The urethral catheter used for the study was a size 8 double lumen French urethral catheter inserted in a sitting position. Normal saline solution at room temperature with filling rate at 70 mL/min for cystometrogram and at 2.0 mL/min for urethral profile pressure was used.

Transvaginal pelvic reconstructive surgery for site-specific repair was accomplished under general or regional anesthesia. The surgical procedure occurred in the following manner: vaginal hysterectomy, anterior colporrhaphy with or without transvaginal mesh (TVM) implantation, sacrospinous ligament fixation (SSF), posterior colporraphy and mid-urethral sling insertion when indicated. TVM that provided anterior support such as Perigee^10^, and Avaulta Anterior (Avaulta A)^11^ underwent SSF for apical suspension. SSF was fixed on the right unilateral side via the posterior approach^12^. No additional support was done for TVM that provided anterior and apical support such as Prolift anterior and posterior (Prolift T)^13^, Elevate Anterior/Apical (Elevate A)^14^, and Uphold. Concomitant MUS (Transobturator tape) was offered to patients with higher score in question #3 (Urine leak related to physical activity, coughing or sneezing) than #2 (Urine leakage related to the feeling of urgency) on UDI-6. Patients offered with MUS must have a post-void residual (PVR) urine <20% the voided volume. Cystoscopy to evaluate integrity of the lower urinary tract was performed in all patients who had TVM and MUS inserted. All patients were counseled on the surgical procedures and informed on potential benefits and possible complications during and following surgery. Risk of mesh and MUS related complications, e.g., mesh erosion, chronic pelvic pain, dyspareunia, infection, and the possibility of needing an additional procedure for mesh removal or trimming in case of mesh complications, were included in the counseling. Patients made an informed decision as to whether to have mesh augmented surgery or native tissue repair and/or addition of the MUS procedure.

Post-operatively, Foley catheter was placed for 24 hours prior to removal. PVR urine volume was then checked every 4 hours employing the bladder scan (BVI 3000; Diagnostic Ultrasound Corp., Bothell, WA). For those with PVR urine volume of >150 mL or >20% the voided volume, sterile intermittent catheterization was done. Moreover, when the ideal PVR, which is <20% the voided volume, cannot be attained in 3 days time, patients were taught clean intermittent self-catheterization. Anti-muscarinic medication was given for patients with symptoms of overactive bladder but were stopped a week prior to the urodynamic study. Topical estrogen per vagina was given to postmenopausal patients unless otherwise contraindicated.

**Figure 1:**
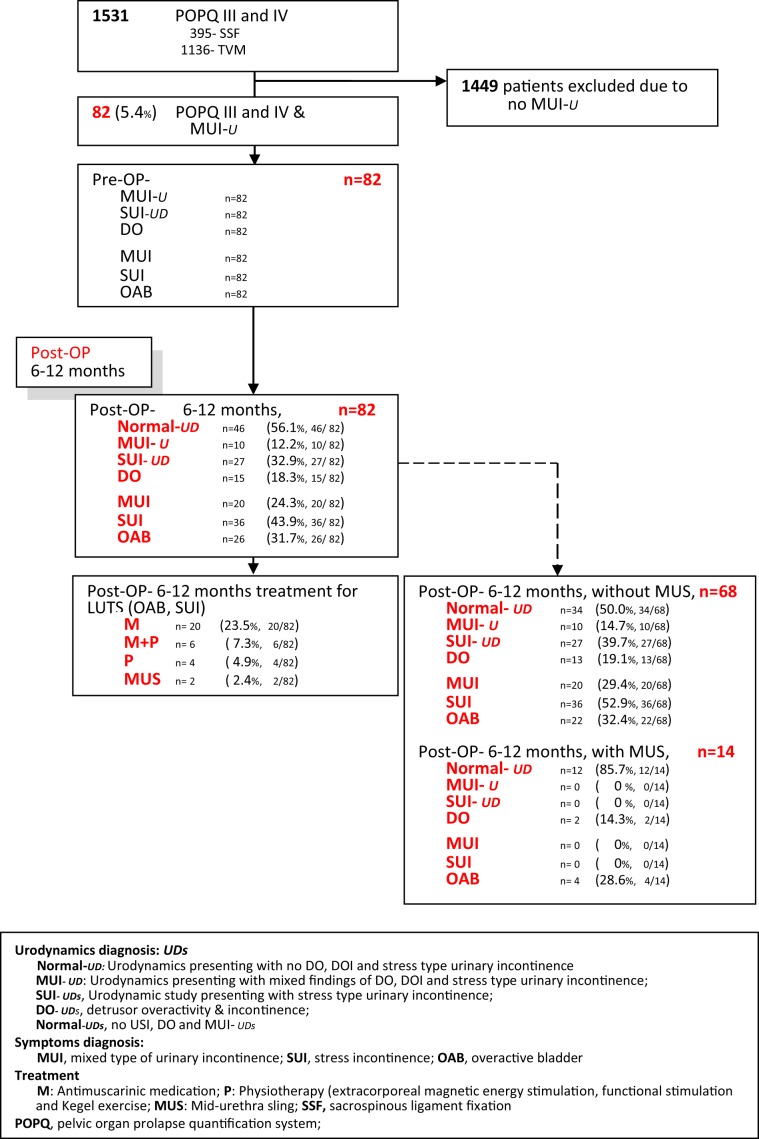
Flow Chart.

Post-operative follow-up followed the institutional protocol. Evaluations were schedule at the following time: 1 week, 1 month, 3 months, 6 months, and annually. During assessment, it included the following: history, subjective complains, pelvic examinations, 3-day voiding diary, measurement of PVR via sterile catheterization, and answering the validated subjective questionnaires. The use multichannel urodynamic study was done at 6 months to 1 year (Fig. 1).

MUI-U defined through urodynamic study as mixed urinary incontinence with findings of both of DO/detrusor overactivity incontinence (DOI) and stress urinary incontinence (SUI-_UD_). DO was defined as spontaneous or provoked involuntary detrusor contraction during filling cystometry producing a waveform pattern of variable duration and amplitude on cystometrogram^15^. SUI-_UD_ defined as involuntary leakage of urine during increased abdominal pressure on filling cystometry regardless of detrusor contraction. Occult SUI-_UD_ was considered when patients had urine leakage when the prolapse was reduced. Bladder outlet obstruction (BOO) was diagnosed when peak flow rate (Q_max_) < 15 ml/s and detrusor pressure at maximal flow (P_det_Q_max_) > 20 cm H_2_O in conjunction with a high clinical suspicion of obstruction^16^.

The main outcome measures were 1) Objective cure- the absence of involuntary detrusor contraction on filling cystometry and no demonstrable leakage of urine during increased abdominal pressure, and 2) Subjective cure- having an assessment index score of <1 on UDI-6 question #2 (Urine leakage related to the feeling of urgency) and #3 (Urine leak related to physical activity, coughing or sneezing).

### Statistical analysis

The use of descriptive statistics was applied for patient demographics and perioperative data. The Fischer’s exact test was used when the assumption of the chi-square test was violated (i.e. when more than one cell had an expected count of <1 or >20% of the cells with expected count of <5). All statistical tests were two-sided. A p value of <0.05 was considered statistically significant. All statistical methods were performed using the Statistical Packaged for Social Sciences (SPSS version 17, Chicago IL, USA).

## Results

There were 1531 women with advanced POP who underwent vaginal PRS with or without MUS insertion. Of these, 82 patients (5%) were diagnosed to have MUI-U and were included in the study. From the inclusion patients, 14 of them had PRS with concomitant MUS insertion while 68 had PRS alone. Post-operatively, 1 year data showed objective cure of MUI-U at 56% (46/82) and subjective cure at 54% (44/82). Persistence of MUI-U was seen in 12% (10/82), SUI-_UD_ in 33% and DO in 18% (15/82) of patients (Fig. 1). The rate of cure for SUI-_UD_ was 67% (55/82) objectively and 59% (48/82) subjectively. Cure for DO was objectively achieved in 82% (67/82) and subjectively in 68% (56/82) of the patient population. Prolapse was corrected in 96% (679/82) of the patients (Table 1).

**Table 1:** Baseline characteristic of 82 MUI patients undergoing with extensive pelvic reconstructive surgery.

	MUI-U and POPQ ≧ stage III,	n = 82
Mean age (year)	65.1 ± 9.8	(58.3–72.6)
Median parity	2	(1–5)
Mean BMI (kg/m^2^)	25.2 ± 3.6	(24.3–26.5)
Prior pelvic surgery	8	(9.7%)
TAH	4	
VH	2	
LH	1	
Colon cancer	1	
**Medical disease**		
Diabetes mellitus	19	(23.1%)
Hypertension	32	(39.0%)
Brest cancer	3	(3.7%)
CVA (Stoke)	2	(2.4%)
Parkinsonian	2	(2.4%)
Post-menopause	71	(86.6%)
**Pre-OP POPQ**		
stage III	49	(59.8%)
stage IV	33	(40.2%)
**Post-OP POPQ, 1**^**st**^ **year**		
stage 0	71	(86.6%)
stage I	8	(9.8%)
stage II	3	(3.6%)
Mean operating time (min)	73.1 ± 10.1	(66.5–82.6)
Mean intraoperative blood loss (ml)	105.1 ± 45.9	(68.3–131.2)
Mean hemoglobin difference (g/dl)	1.1 ± 0.9	(0.3–1.9)
Mean post-OP hospital stay (days)	4.1 ± 0.8	(3.7–4.6)
Median period of follow-up (months)	49.5 ± 32.2	(12.1–77.9)
**Complications**		
Mesh exposure, vagina	2 (Perigee × 2)	
Infection	1 (Antibiotic control)	
Voiding dysfunction, post-OP	1 (Pre-OP underactivity)	
Other complications	1 (Stroke 4 year after OP)	
Obj. cure, MUI-*U*, 1^st^ year	56.1%, (46/82)	
Subj. cure, MUI, 1^st^ year	53.6%, (44/82)	
Obj. cure, SUI-*UD*, 1^st^ year	67.1%, (55/82)	
Subj. cure, SUI, 1^st^ year	58.5%, (48/82)	
Obj. cure, DO, 1^st^ year	81.7%, (67/82)	
Subj. cure, OAB, 1^st^ year	68.3%, (56/82)	
Obj. cure, POP, 1^st^ year	96.3%, (679/82)	
Subj. cure, POP, 1^st^ year	93.9%, (77/82)	

Baseline characteristic of 82 MUI patients undergoing extensive pelvic reconstructive surgery.

Data are listed as mean ± standard deviation with 95% CI in parentheses or number with percentage within parentheses.

BMI, body mass index; TAH, total abdominal hysterectomy; VH, vaginal hysterectomy; LH, laparoscopic hysterectomy; TOT, trans-obturator tape; UUI, urgency urinary incontinence; Obj, objective; Subj, subjective;

MUI-*U*: Urodynamic study presenting with mixed findings of DO, DOI and stress type urinary incontinence; SUI-*UD*, Urodynamic study presenting with stress type urinary incontinence; DO, detrusor overactivity & incontinence; MUI, mixed typed urinary incontinence; OAB, overactive bladder; POP, pelvic organ prolapse.

Baseline demographic data were shown in Table 1. Eighty-seven percent of the patient population belonged to post-menopausal women, with a mean age of 65.1 ± 9.8 years old. The population concurrently had a median parity of 2. There were 8 patients (10%) with prior pelvic surgeries. The most common medical disease noted was hypertension (39%) followed by diabetes mellitus (23%). Pre-operatively, 49 (60%) patients had stage III prolapse and 33 (40%) had stage IV prolapse. The surgical procedure took 73.1 ± 10.1 minutes with intraoperative blood loss of 105.1 ± 45.9 ml. The mean duration of hospital stay was 4.1 ± 0.8 days. Post-operative complications included infection, voiding dysfunction, mesh exposure, and stroke. The mean post-operative follow-up period was 49.5 ± 32.2 months.

Comparison of pre- and post-operative urodynamic study findings on clinical outcomes from 6 months to 1 year were shown in Table 2. Post-operative patients with MUI-U had significantly improved (p < 0.001). The number of patients with normal urodynamic findings significantly increased (p < 0.001) as well. Independent findings of DO/DOI, SUI-_UD_, and BOO had also significantly improved (p < 0.001). Noticeably, the significant improvement of SUI-_UD_ (p < 0.001) and 1-hour pad test was more pronounced in patients with concomitant MUS insertion when compared to PRS alone. Significant changes in urodynamic parameters were observed. Maximum urinary flow (Qmax) and cystometric capacity (CC) significantly increased, while post-void residual urine (RU), maximum urethral closure pressure (MUCP), functional urethral length (FUL) and detrusor pressure at maximum flow (Dmax) significantly decreased. Symptoms of urgency and SUI significantly improved.

**Table 2:** Comparison of pre and post-operative (6 months to 1 year) clinical outcomes.

	Pre-OP, n = 82	Post-OP, n = 82	*p*-value	Post-OP, subgroups	*p*-value (between subgroup)
				without MUS, n = 68	
				with MUS, n = 14	
Qmax	15.1 ± 9.2	20.1 ± 5.7	<0.001		
	(11.7–19.7)	(16.8–23.9)			
				20.4 ± 4.9	0.623
				(17.1–23.7)	
				19.7 ± 4.3	
				(16.9–22.7)	
RU	90.1 ± 61.6	36.2 ± 19.9	<0.001		
	(52.6–142.1)	(23.6–58.2)			
				35.2 ± 17.5	0.377
				(23.3–49.2)	
				37.9 ± 18.5	
				(25.6–55.7)	
CC	262.7 ± 141.3	327.5 ± 87.4	<0.001		
	(196.3–316.2)	(272.7–373.2)			
				310.5 ± 76.2	0.173
				(271.6–351.3)	
				336.4 ± 82.1	
				(282.9–376.3)	
MUCP	92.8 ± 42.6	75.5 ± 33.1	<0.001		
	(63.6–118.3)	(57.4–85.1)			
				75.1 ± 22.1	0.215
				(55.1–86.7)	
				76.5 ± 32.0	
				(58.6–79.2)	
FUL	23.5 ± 5.5	20.1 ± 4.2	0.015		
	(19.8–26.4)	(18.6–22.5)			
				19.9 ± 3.1	0.682
				(18.0–21.5)	
				20.5 ± 3.3	
				(18.8–22.9)	
Dmax	28.1 ± 13.4	16.9 ± 5.1	<0.001		
	(19.2–35.9)	(13.5–19.1)			
				16.5 ± 5.8	0.209
				(13.1–19.8)	
				17.2 ± 5.1	
				(13.5–20.7)	
**UDs** **diagnosis**	**Pre-OP, n** = **82**	**Post-OP**, **n** = **82**	***p*-value**	**Post-OP, subgroups**	***p***-value (between subgroup)
				**without MUS, n = 68**	
				**with MUS, n = 14**	
MUI-*U*	82	10	<0.001		
	68			10	0.198*
	14			0	
DO/DOI-*UDs*	82	17	<0.001		
	68			13	0.474*
	14			2	
SUI-*UD*	82	27	<0.001		
	68			27	0.003*
	14			0	
BOO	28	0	<0.001*		
	28			0	x
	0			0	
DU	3	2^++^	0.500*		
	3			2^++^	0.686*
	0			0	
**UDs** **diagnosis**	**Pre-OP, n** = **82**	**Post-OP**, **n** = **82**	***p*-value**	**Post-OP, subgroups**	***p***-value (between subgroup)
				**without MUS, n = 68**	
				**with MUS, n = 14**	
Normal	0	46	<0.001*		
	0			34	0.018*
	0			12	
1 hour pad test	Pre-OP, n = 102	Post-OP, n = 102	*p*-value	Post-OP, subgroups	*p*-value
				without MUS, n = 68	
				with MUS, n = 14	
	18.2 ± 18.5	1.7 ± 3.7	<0.001		
	(3.4–31.2)	(0.2–3.2)			
				2.5 ± 3.2	**0.011**
				(0.6–4.3)	
				0.5 ± 0.2	
				(0.3–0.7)	
**LUTS**	**Pre-OP, n** = **82**	**Post-OP, n** = **82**	***p*-value**	**Post-OP, subgroups**	***p*-value**
				**without MUS, n** = **68**	
				**with MUS, n** = **14**	
Urgency	82	26	<0.001*		
				22	0.526*
				4	
SUI	82	36	<0.001		
				36	0.001*
				0	

Qmax, maximum urinary flow (m/s); RU, postvoid residual urine (ml); CC, cystometric capacity (ml); MUCP, maximum urethral closure pressure (cm H_2_O); FUL, functional urethral length (cm); Dmax, detrusor pressure at maximum flow (cm H_2_O);

MUI-*U*, Urodynamic study presenting with mixed findings of DO, DOI and stress type urinary incontinence; DO/DOI, detrusor overactivity/incontinence; SUI-UD, stress type urinary incontinence at urodynamic test; BOO, Bladder outlet obstruction; DU, detrusor underactivity; LUTS, lower urinary tract symptoms; SUI, stress urinary incontinence.

Data listed as mean ± standard deviation (95% confidence interval).

*Fisher exact test.

^++^SUI-*UDs* with detrusor underactivity.

Subjective evaluation of symptoms through validated questionnaires UDI-6 (p < 0.001), IIQ-7 (p < 0.001), and PISQ-12 (p < 0.001) has shown significant improvement of symptoms (Table 3).

**Table 3:** UDI-6, IIQ-7 and PISQ-12 scores pre and postoperative.

	UDI-6			
	MUI-U & POP		P value	P value*
Pre op, n = 82	11.5 ± 3.3	(9.3–13.5)	<0.001	
Post op 1st year, n = 82	7.8 ± 2.6	(5.7–9.5)		
without MUS, n = 68	8.2 ± 2.3	(6.8–9.5)		<0.001
with MUS, n = 14	5.6 ± 2.2	(4.1–6.8)		
	**IIQ-7**			
	**MUI-U & POP**		**P value**	P value*
Pre op, n = 82	14.0 ± 3.8	(11.6–16.1)	<0.001	
Post op 1st year, n = 82	9.7 ± 3.1	(7.9–11.5)		
without MUS, n = 68	10.1 ± 2.7	(8.4–12.3)		<0.001
with MUS, n = 14	7.9 ± 2.4	(6.3–9.4)		
	**POPDI-6**			
	**MUI-U & POP**		**P value**	P value*
Pre op, n = 82	16.5 ± 4.0	(13.1–18.8)	<0.001	
Post op 1st year, n = 82	10.4 ± 3.1	(8.5–12.7)		
without MUS, n = 68	10.5 ± 2.8	(8.9–12.2)		0.331
with MUS, n = 14	10.2 ± 2.3	(8.8–11.6)		
	**PISQ-12**			
	**MUI-U & POP**		**P value**	P value*
Pre op, n = 24	19.1 ± 4.8	(15.7–22.7)	<0.001	
Post op 1st year, n = 24	25.8 ± 3.2	(23.5–27.9)		
without MUS, n = 21	25.2 ± 2.5	(23.3–27.1)		0.021
with MUS, n = 3	29.5 ± 3.1	(27.3–31.9)		

Data listed as mean ± standard deviation with 95% CI in parentheses.

MUI-U, mixed type of urinary incontinence; POP, pelvic organ prolapse; UDI-6, Urinary Distress Inventory; IIQ-7, Incontinence Impact Questionnaire; PISQ-12, Pelvic Organ Prolapse/Urinary Incontinence Sexual Questionnaire.

Paired-samples t test; P < 0.05 was considered statistically significant.

*p value between with and without MUS.

## Discussion

The study mainly focused on patients with MUI-U associated with advanced prolapse because the study aims to provide objective assessment and evidence of cure. Urodynamic studies may be difficult to use as basis for objective assessment since not all institutions have this equipment. As part of the institutions protocol, a urodynamic study is done in all POP patients pre- and post-operatively. MUI-U occurred in 5% of the advanced prolapse patient population. After vaginal PRS, MUI-U resolved objectively in 56% and subjectively in 54% of the patients. The cure rates of the present study were almost comparable with other published reports. Wolter, *et al*.^17^ demonstrates resolution of MUI in 63% (34/70) of patients after correction of grade 3–4 cystocele with sling insertion. Nguyen and Bhatia^18^ shows 63% (24/38) of patients with resolution of urge urinary incontinence after surgical prolapse repair. Foster, *et al*.^19^ also found resolution of urgency, frequency and urge urinary incontinence in 76%, 55% and 75% of patients after reconstructive or obliterative surgery with or without sling insertion.

It is theorized that the main cause of MUI-U in advanced POP patients is obstruction and weakening of the endopelvic fascia. Restoration of the normal anatomy of the anterior and apical compartment through vaginal PRS led to cure of MUI-U in half of the patient population. The obstruction caused by the POP led to denervation of the bladder from super-sensitized reaction to neurotransmitters^20^, ischemia and hypoxia from distention^21^. The number of muscle contractile units decreases thereby, reducing cell-to-cell propagation of membrane potential^22^ and loss of synchronization^23^ making the bladder overactive and irritable. The weakening of the endopelvic fascia led to loss of tension, urethral distortion, and ability to maintain the bladder in its correct position for detrusor control and continence function, allowed incontinence to occur^24^.

The significant improvement of DO (82%), OAB (68%) symptoms, and voiding function noted after vaginal PRS showed the importance of relieving mechanical obstruction for restoration of normal bladder function^25^. Urodynamic study objectively shows significant increase in Qmax and decrease in RU and Dmax. Similarly, Basu and Duckett^25^ shows improvement of OAB in 53% of women after prolapse surgery with significant improvement in flow rates. Kim, *et al*.^26^ also demonstrates improvement of urge incontinence and urinary frequency in 88% and 74% respectively, after surgical repair of stage 3 to 4 POP.

A proportion of the patient population had persistence of DO at 18% and OAB in 32%. Other inherent factors such as age ≥ 66 years, neurologic e.g. Parkinson’s disease and cerebrovascular accidents, pre-operative (MUCP ≥ 60 cm H_2_O, maximum flow rate < 15 ml, Dmax ≥ 20 cm H_2_O), and PVR > 200 ml^27^ cause persistence of DO after PRS. Concomitant sling surgery is also a known factor to increase the rate of DO or OAB^17^. The present study, however, did not show significant increase in DO or symptoms of urgency to the MUS group. The relatively small number of patients who had concomitant MUS insertion could not reflect the true outcome of DO.

The discrepancy in cure rates of DO and OAB was a result of the difference in physician - patient perception of cure. Similarly, Wolter, *et al*.^17^ shows 35% of patients with MUI had DO on UDS while 24% had no symptoms of OAB but had DO. Gilleran, *et al*.^28^ looked into urodynamic findings before and after prolapse reduction, showing that the presence of DO remained constant. A substantial portion of patients had OAB but had no evidence of DO on UDS. Nonetheless, anticholinergic medications were given to these patients for symptom improvement (24% anti-muscarinic and 7% combined with physiotherapy).

Reestablishment of the apical compartment lead to continence in 60% (41/68) of patients and the addition of transobturator tape (TOT) increased continence rates to 100%. Correspondingly, Stumm, *et al*.^29^ demonstrates continence in 26–43% of patients with MUI after prolapse surgery and the addition of TOT increased rates to 63–65%. Wolter, *et al*.^15^ shows a 92% cure rate of SUI after anterior POP repair and sling insertion in patients with MUI with significant risk for post-operative urgency.

MUI-U patients are not offered concomitant anti-incontinence procedures routinely to reduce surgical complications and voiding dysfunction, unless SUI symptoms are predominant. Lo *et al*. reported that concomitant MUS insertion along with advance POP surgery was a contributing factors for increasing the risk of post-operative voiding dysfunction at 3.12 times in odds ratio^30^. It is advisable to await effects of POP surgery. A large cohort prospective study by Lensen, *et al*.^31^ demonstrated a subjective cure of 39% for pre-existing SUI and 42% with urge urinary incontinence treated with POP surgery alone. Borstad’s similarly show 27% of women cured of SUI after surgical repair of POP^32^. The persistence of SUI in 40% (27/68) of the patients without MUS is likely due to having MUCP < 60 cm H_2_O and FUL < 2 cm together with overt SUI and advanced POP^33^.

### Strengths and limitations

The study is limited by its’ retrospective study design, involvement of one surgeon, and exclusion of subjective symptoms. Strengths of the study include a large number of patients who underwent a standardized preoperative evaluation protocol using standard ICS recommendations, standardized operative procedures, documentation utilizing standard pro forma, and continuous long-term patient follow-up.

## Conclusion

In conclusion, patients with advanced POP presenting with MUI-U can be managed with vaginal PRS. The restoration of the anatomical defect provides improvement of symptoms subjectively at 54% and objectively cures at 56%. Giving anti-cholinergics to OAB symptoms are helpful once obstruction is relieved. Doing concomitant MUS can be an effective and viable option for SUI predominant MUI after thorough patient counseling.
